# Risk Assessment of Heavy Metals in Sediment Samples from the Mae Chaem River, Chiang Mai, Thailand

**DOI:** 10.3390/toxics11090780

**Published:** 2023-09-14

**Authors:** Sawaeng Kawichai, Tippawan Prapamontol, Teetawat Santijitpakdee, Susira Bootdee

**Affiliations:** 1 Research Institute for Health Sciences (RIHES), Chiang Mai University, Chiang Mai 50200, Thailand; sawaeng.k@gmail.com (S.K.); teetawat_san@cmu.ac.th (T.S.); 2 Faculty of Science, Energy, and Environment, King Mongkut’s University of Technology North Bangkok (Rayong Campus), Rayong 21120, Thailand; susira.b@sciee.kmutnb.ac.th

**Keywords:** heavy metals, enrichment factor, pollution load index, hazard quotient

## Abstract

Heavy metals are significant environmental pollutants that are recognized as posing a potential health hazard to human beings. We investigated the concentrations of the heavy metals As, Cd, Cr, Cu, Ni, Pb, and Zn in surface sediments collected from the Mae Chaem River in Chiang Mai, Thailand, during the dry season in 2021. The mean concentrations of heavy metals in sediments were, in decreasing order, Zn > Cr > As > Pb > Ni > Cu > Cd. The mean values of As, Cd, Cr, and Cu were determined to be 32.5 ± 18.3, 0.33 ± 0.07, 45.8 ± 11.9, and 21.9 ± 7.42 mg Kg^−1^, respectively. These levels are higher than their standard levels in Thailand, namely 10.0, 0.16, 45.5, and 21.5 mg Kg^−1^, respectively. Principal component analysis (PCA) revealed that the primary origins of heavy metal contamination are predominantly attributed to residential settlements and agricultural areas. The hazard quotient (HQ) was used to estimate the non-carcinogenic risk of exposure to heavy-metal-bound surface sediments for both children and adults. The results showed that the HQ values for both groups were less than 1.0 (HQ < 1.0), indicating no risk. Moreover, assessment of the long-term risk for ingestion of toxic metals indicated no risk (<10^−6^) based on the lifetime cancer risk (LCR). However, the LCR values of As and Cr were 5.3 × 10^−6^ and 2.5 × 10^−6^, respectively, demonstrating the most elevated LCR among the hazardous metals in terms of children’s exposure. Therefore, it is possible that children living in agricultural areas and participating in activities around the study area may be exposed to elevated concentrations of As and Cr.

## 1. Introduction

As a critical feature of the age of globalization, chemical fertilizers and pesticides are a mainstay of modern agricultural production and are used to increase crop yields [1] and control weeds and insects [2]. The global use of pesticides was 0.2 million tons in the 1950s and more than 5 million tons in the 2000s [3]. Chemical fertilizers and pesticides increase the quantities of heavy metal contaminants in the environment [4,5]. Dangerous heavy metal groups are used as catalysts to produce fertilizers [6]. Previous research has shown that the heavy metals in fertilizers and pesticides appear to produce heavy metal contaminants in their products [5,7]. The fertilizers were shown to release several heavy metals, including chromium (Cr), cadmium (Cd), copper (Cu), zinc (Zn), nickel (Ni), manganese (Mn), and lead (Pb) [8,9,10]. On the other hand, manure fertilizer was found to emit Zn, Cu, Ni, Pb, Cd, Cr, arsenic (As), and mercury (Hg) [11,12,13]. Moreover, the average concentration of Cd in phosphate, nitrogen, and manure fertilizer was found to range from 0.1 to 170, 0.05 to 8.5, and 0.3 to 0.8 mg Kg^−1^, respectively. Similarly, the Cu concentrations varied from 7.0 to 225, 2 to 1450, and 6.6 to 350. Zn concentrations were found to be within the range of 50 to 1450, 1.0 to 42, and 15 to 250. Finally, Pb concentrations were measured to be from 1.0 to 300, 1.0 to 1.5, and 2.0 to 6.0 [14]. The three types of pesticides that are most frequently used globally are fungicides, insecticides, and herbicides. These are sources of heavy metals such as Cu, Zn, Cd, Pb, and As [15,16]. According to a previous study, heavy metals including Zn, Pb, and Cd are present at high concentrations in pesticides and herbicides [17].

According to the World Health Organization (WHO), heavy metals may be carcinogenic [18], and potential exposure comes as a result of ingesting, drinking, or interacting with heavy metal residue in plants, animals, and water [19]. Heavy metals are also present in the air, and contaminated air is also a great health concern [20,21]. Cr could cause nephritis and ulceration [22], while Ni could cause genotoxicity and lung cancer [23]. Arsenic (As) has been identified as a potential carcinogen and has been associated with reproductive and developmental abnormalities [24]. On a global scale, the number of soil pollution sites has exceeded 10 million. The data related to soil pollution revealed that more than 50% of the reported sites demonstrated evidence of heavy metal contamination. A study conducted in the European Union, the United States, and Australia revealed the presence of heavy metal contamination at 37% of 80,000 sites, over 70% of 100,000 sites, and more than 60% of 50,000 sites, respectively [25]. The results of these studies revealed that heavy metals from fertilizer and pesticide sources could be retained in soil, crops, sediment, and aquatic animals. By comparing the concentrations of heavy metals in soil, aquatic animals, sediment, and water samples containing used and unused fertilizer, it was shown that samples with unutilized fertilizer had higher concentrations of heavy metals than the utilized fertilizer and pesticide samples [5,26,27]. Governments of several countries were concerned by heavy metal contamination that could affect human health. They regulated policy standards for heavy metal concentrations, specifically for the control of agricultural soils, water, and sediment [25,28]. Southeast Asian countries realized the importance of heavy metal contamination in sediment from agriculture, and there have been numerous studies about heavy metal contamination in sediment. For instance, researchers from Vietnam, Thailand, Malaysia, the Philippines, and Indonesia examined the advantages and disadvantages of using river sediment for agriculture in their respective countries [29]. Similarly, Malaysian researchers conducted a study to determine the sediment profile of 210 Pb, Pb, U, and Th in the Sultan Abu Bakar Dam, with such contamination being the result of soil erosion in the highland agricultural region of the Cameron Highlands, Malaysia [30].

The findings in Thailand demonstrated that sediments along the eastern coast of the Gulf of Thailand were contaminated with heavy metals due to various anthropogenic sources, including agriculture, fisheries, tourism, industry, and urban areas. Analysis of the data revealed that the concentrations of Pb and Cu in the sediments exceeded the minimum requirements of the marine and coastal sediment quality standards established by the Thailand Amendment [31]. Contamination with Pb, Zn, and As has become major in the southern part of Thailand near abandoned mines located in the granite mountains [32]. Moreover, the Khuan Khi Sian wetland, a district of Phatthalung Province, was investigated, and the contaminant concentration levels of Pb, As, and Cd in the dry and wet seasons were reported. The data showed that the highest contaminant concentrations in both seasons were Pb, As, and Cd. The high levels of arsenic compounds found in sediment could be related to the direct transformation of chemical fertilizers into agricultural wastewater which then flows from land to wetland [33].

The main objectives of this study were to determine the levels of heavy metals in the surface sediment of the Mae Chaem River in Chiang Mai and to evaluate the potential implications for health risks associated with heavy metal exposure.

## 2. Materials and Methods

### 2.1. Study Areas and Sample Collection

This study was conducted in the Mae Chaem District in southwest Chiang Mai Province, Thailand, specifically focusing on the Mae Chaem River, which is a significant river in the area. The area is characterized by its topographical features, mostly consisting of elevated topography and surrounding land used for agriculture. The water from this river is utilized for both consumption as drinking water and agricultural purposes. The Mae Chaem (Chaem River) watershed (18°06′–19°100′ N and 98°04′–98°34′ E) is located in the Mae Chaem District, Chiang Mai Province, of upper northern Thailand. It is a significant upper tributary subbasin of the Ping River, the main tributary of central Thailand’s Chao Phraya River. Mae Chaem District had 59,636 residents in 2021. The Karen and Lua ethnic groups dwell primarily in highland villages located over 1000 m above sea level (m.a.s.l.) and are distributed among numerous small villages, with some communities reaching higher elevations. In contrast, ethnically northern Thai (Khon Muang) villages are generally concentrated in lowland areas below 600 m.a.s.l. In addition, it was found that most of the population had a career in agriculture. The Mae Chaem District is the most agricultural area of Chiang Mai. The agriculture pattern is comprised of the production highland (above 1000 m.a.s.l.) and includes significant increases in highland cash crops such as maize and a rice cultivation area of about 20,000 acres. Therefore, in areas where agricultural chemicals are still widely used, in order to evaluate the level of heavy metals from the usage of fertilizer and pesticides in Mae Chaem District, Chiang Mai Province, Northern Thailand, we collected sediment samples near agricultural regions for this study. This area was selected as it is isolated from the urban city and has a significant number of wild areas and agricultural fields, which may contain heavy metals in the soil.

**Figure 1:**
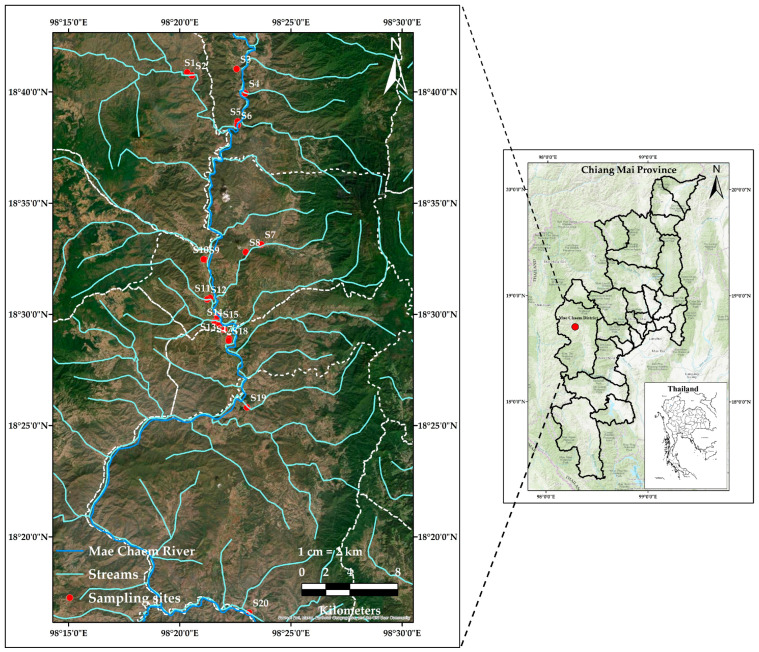
Map of the study area and sampling site locations.

Figure 1 illustrates the geographical coordinates of the sites, where a total of twenty sediment samples were collected during the month of May in the year 2021. Table S1 provides the land-use activities, watershed information, and site code location of the Mae Chaem River. Samples of sediment were collected from various depths, particularly between 10 and 20 cm, with the specific depth being determined based on the state of the substance at that point. The sediment samples were taken, stored in clean zip-lock plastic bags, and then transported to the laboratory. The samples were air-dried before being placed in a hot air oven at 80 °C for 6 h and air-dried until the mass was stable at room temperature (25 ± 2 °C). All of the samples were passed through a nylon sieve (1/56 mm) with a standard testing sieve to remove coarse particles larger than 63 µm, and this size was found to be the best size for metal analysis [34], and then placed in a polyethylene plastic bag. Finally, all of the sediment samples were kept in a freezer at a temperature of −20 °C until the analysis.

### 2.2. Sample Analysis

Heavy metals in the sediment samples were determined at the Research Institute for Health Sciences, Chiang Mai University, Thailand. Initially, 0.15 g of each dried sample was placed in a sealed Teflon vessel and digested in a microwave digestion system with 800 W power at 210 °C for 20 min with a mixed acid concentration of 10 mL of HNO_3_ and 2 mL of HCl. After cooling to room temperature, the digested solution was transferred to a 25 mL volumetric flask and diluted with deionized water. The suspended material was filtered through 0.45-µm filters into a 50 mL test tube. The digested samples were determined to have heavy metal contents using inductively coupled plasma optical emission spectrometry (ICP-OES, Agilent 5800 series, Santa Clara, CA, USA). The measurement of heavy metal levels in each sample was conducted using concentration and absorbance calibration curves. A straight line with *r* > 0.999 was used to statistically analyze the data. The analyses of As, Cd, Cr, Ni, Pb, Cu, and Zn were performed in triplicate using standard reference material (SRM) 1648a to ensure the accuracy of the procedure. The results relevant to the percentage recovery of heavy metals demonstrated a range of 48.7% to 117.1%, as shown in Table S2. Furthermore, the precision error that accompanied these measurements was found to be below 3%, as shown in Table S2.

### 2.3. Pollution Assessment of Heavy Metals

#### 2.3.1. Geo-Accumulation

The degree of heavy metal contamination can be determined by calculating the geo-accumulation index (I*_geo_*), which Muller developed in 1969 to quantitatively evaluate pollution and indicate heavy metal accumulation in sediment [35]. The geo-accumulation index (I*_geo_*) is widely used to describe the contamination condition of surface sediments in aquatic environments. It can also be used to assess the level of heavy metal contamination (I*_geo_*) [36,37]. The geo-accumulation index (I*_geo_*) was calculated using the equation as follows:

(1) $$I_{\mathrm{geo}}={\mathrm{Log}}_{2}[\frac{C_{n}}{1.5\times B_{n}}]$$

where C_n_ is the heavy metal concentration in the sediment sample (mg Kg^−1^) and B_n_ is the geochemical background value of element “n”. Taylor’s referenced heavy metal background values, which include As, Cd, Cr, Ni, Pb, Cu, and Zn were 1.8, 0.2, 100, 55, 75, 12.5, and 70 mg Kg^−1^, respectively [38]. Here, 1.5 is used for the background matrix correlation factor and the concentration categories classified by lithogenic effect [35], based on a geo-accumulation index (I*_geo_*) divided into seven classes, as shown in Table S3.

#### 2.3.2. Contamination Factor

The contamination factor (CF) was determined to be the most simple and effective method for monitoring the level of heavy metal contamination, and is calculated using the following formula (Equation (2)):

(2) $$\mathrm{CF}=\frac{C_{\mathrm{sample}}}{C_{\mathrm{background}}}$$

where C_sample_ is the concentration of heavy metals in a sediment sample, and C_background_ is the mean concentration value of the metal in the natural background. The background concentration standards for As, Cd, Cr, Ni, Pb, Cu, and Zn were set by the Chinese Soil Environmental Quality Standard (GB 15618-1995) and were 15, 0.2, 90, 26, 35, 35, and 100 mg Kg^−1^, respectively. The ratio between the measured concentration of heavy metal in the sediment samples and the natural abundance of metal can be provided as an index. As a result, CF levels were then divided into four grades for pollution assessment of each metal over a period of time on a scale ranging from 1 to 6 [39], as shown in Table S3.

#### 2.3.3. Pollution Load Index

The pollution load index (PLI) is used to assess the quality of sediments. It represents the number of times the heavy metal concentration in the sediment exceeded the background concentration, causing the sediment condition to deteriorate due to the accumulation of heavy metal [40,41]. In this index, the PLI is defined as the ^n^th root of the multiplication of the contamination factor (CF) of the target heavy metal and is calculated as follows (Equation (3)):

(3) $$\mathrm{PLI}={\left({\mathrm{CF}}_{1}\times {\mathrm{CF}}_{2}\times \ldots \ldots \times {\mathrm{CF}}_{n}\right)}^{\frac{1}{n}}$$

where CF_1_ represents the CF value of metal 1, CF_2_ represents the CF value of metal 2, CF_3_ represents the CF value of metal 3, CF_n_ represents the CF value of metal n, and n represents the total number of metals studied in the samples. PLI values are interpreted at three levels. A PLI value of 0 indicates excellent, a value of 1 indicates only background level presence, and a value greater than 1 indicates progressive deterioration of the site [42].

### 2.4. Health Risk Assessment of Heavy Metals

#### 2.4.1. Non-Carcinogenic Risk Assessment

The non-cancer-causing risk was calculated using the hazard quotient (HQ), which is a tool proposed by the US-EPA to measure the potential non-cancer-causing health risks of metals in soil for organisms. The hazard quotient (HQ) is a calculated ratio between the exposure level of the average daily intake (ADI) of metal in sediment and the reference doses (RfD) for that substance obtained over an equal exposure period [43,44,45]. The HQ value can be calculated by using Equation (4), as presented below.

(4) $$\mathrm{HQ}=\frac{\mathrm{ADI}}{\mathrm{RfD}}$$

The reference dose (RfD) for a heavy metal in sediment, as obtained from the regional screening level (RSL) summary table from 2022 [46], is denoted by RfD and expressed in milligrams per kilogram (mg Kg^−1^). This information may be found in Table S4. HQ values less than 1.0 indicate that the metal under consideration is unlikely to have a negative effect on health, but HQ values greater than 1.0 indicate a significant potential for health effects. Equation (5) can be used to estimate the ADI by ingestion [43,44,45]. The specific values for these parameters can be found in Table S4, as indicated by references [47,48].

(5) $$\mathrm{ADI}=\frac{C\times \mathrm{IR}\times \mathrm{EF}\times \mathrm{ED}}{\mathrm{BW}\times \mathrm{AT}}\times 10^{-6}$$

The variable ADI represents the average daily intake of metals in soil, measured in milligrams per kilogram per day. Meanwhile, the variable C denotes the concentration of metals in sediment, measured in milligrams per kilogram. The soil intake rate (IR) represents the amount of soil ingested per day in milligrams. The exposure frequency (EF) refers to the number of days per year during which exposure occurs. The exposure duration (ED) represents the length of time in years over which exposure takes place. The body weight (BW) of the exposed group is measured in kilograms. Lastly, the average time (AT) refers to the duration of exposure in days. The values corresponding to these parameters are presented in Table S4.

#### 2.4.2. Carcinogenic Risk Assessment

For carcinogenic risk, the toxic metal concentration at which the risk is acceptable is defined by the International Agency for Research on Cancer (IARC) [49]. The assumption is that if the carcinogenic effect results from chronic consumption of a chemical in soil or sediment, then the exposure unit may be determined by the long-term average concentration of ingestion from the contaminated soil or sediment in a river.

The lifetime cancer risk (LCR) refers to the risk of an individual developing cancer due to prolonged exposure to a carcinogenic metal. This estimation is based on the potential of the toxic metal to cause cancer over a long period of time. The estimation was conducted with Equation (6), as presented below. Based on the LCR categories, a value of LCR less than 10^−6^ is deemed insignificant, whereas an LCR level exceeding 10^−4^ is seen as posing a significant risk. The range of acceptable or achievable risk is denoted by a probability of incidence between LCR 10^−6^ and 10^−4^.

(6) $$\mathrm{LCR}=\mathrm{ADI}\times {\mathrm{CSF}}_{\mathrm{oral}}$$

where CSF_oral_ represents the cancer slope factor (expressed as per mg Kg^−1^ day^−1^) and was derived from the RSL Summary Table (US-EPA, 2022), presented in detail in Table S5.

### 2.5. Statistical Analysis

The study evaluated data referring to the concentrations of heavy metals. Utilizing Student’s *t*-test, statistical analysis evaluates the data. Prior to conducting Pearson correlation analysis, a normality test was conducted using IBM SPSS 19.0 Statistics (SPSS Inc., Chicago, IL, USA), with a significance level of 0.05. Principle component analysis (PCA) and factor analysis have been used to identify the major sources of heavy metal distribution in the Mae Chaem River.

## 3. Results and Discussion

### 3.1. Heavy Metal Concentrations

The data shown in Table 1 demonstrate the mean levels of heavy metal concentrations observed in the present study. The concentrations of As, Cd, Cr, Ni, Pb, Cu, and Zn in the sediment sample were observed to be in the range from 14.1 to 95.1 mg Kg^−1^, 0.20 to 0.46 mg Kg^−1^, 27.3 to 70.8 mg Kg^−1^, 13.6 to 38.1 mg Kg^−1^, 12.1 to 43.0 mg Kg^−1^, 8.75 to 36.8 mg Kg^−1^, and 36.7 to 117 mg Kg^−1^, respectively. The mean concentrations (Mean ± S.D.) of these metals were 32.5 ± 18.3 mg Kg^−1^, 0.33 ± 0.07 mg Kg^−1^, 45.8 ± 11.9 mg Kg^−1^, 24.0 ± 6.56 mg Kg^−1^, 27.2 ± 7.42 mg Kg^−1^, 21.9 ± 7.50 mg Kg^−1^, and 60.9 ± 19.3 mg Kg^−1^, respectively. Table 1 presents the observed maximum concentrations of As, Cd, Cr, Ni, Pb, Cu, and Zn in sediments at various sites. The concentrations were found to be particularly elevated at the following locations: S4 (As = 95.1 mg Kg^−1^), S19 (Cd = 0.46 mg Kg^−1^), S14 (Cr = 70.8 mg Kg^−1^), S10 (Ni = 38.1 mg/kg), S19 (Pb = 40.1 mg Kg^−1^), S2 (Cu = 36.8 mg Kg^−1^), and S7 (Zn = 117 mg Kg^−1^). The study determined the relative concentrations of sediment in the Mae Chaem River to be in the following order: Zn displayed the highest concentration, followed by Cr, As, Pb, Ni, Cu, and Cd. The mean concentrations of As, Cd, and Cr exceeded the standards established by the Pollution Control Department (PCD) of Thailand. Nevertheless, with one exception of Ni and Cr, the mean amount of heavy metal concentrations in the surface sediments of the Mae Chaem River in Chiang Mai, Thailand, were found to be within the recommended criteria set by the World Health Organization (WHO).

Additionally, it is important to point out that the concentrations of As, Cd, Cr, Ni, Cu, and Zn observed in the present study were comparatively lower than the values reported in previous studies conducted on the Xiangjiang River, Majiagou River, and Yunliang River in China [50,51]. Furthermore, it is interesting that the concentrations of heavy metals observed in the current study were found to be higher compared with the values reported in previous studies carried out in the Batin River in Turkey and the Shitalakhya River in Bangladesh [44,52]. The results obtained from previous studies conducted on heavy metal concentrations in river sediments in Thailand [53,54,55] demonstrate that the heavy metal concentrations observed in this study during the dry season were 1–4 times higher than what was reported for the Lam Plai Mat River, Chao Phraya River, and Mae Klong River in Thailand, as shown in Table 2.

**Figure 2:**
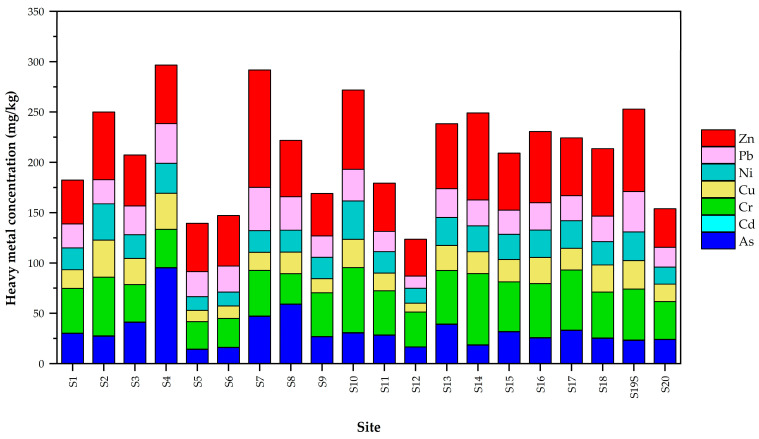
Spatial distribution of heavy metals in the sediment of the Mae Chaem River.

The sediment samples collected along the Mae Chaem River showed higher levels of Cu, Zn, Ni, and Cr in community areas (S2, S7, S10, and S14). However, the concentrations of As, Cd, and Pb were predominantly found in sediment samples obtained from agricultural areas (S4 and S19). The surface sediment of the Mae Chaem River demonstrated the highest amounts of heavy metals in locations S4 and S7, corresponding to the community and agricultural areas, respectively. In agricultural areas, the major heavy metal seen in S4 was arsenic (As), whereas in community areas, zinc (Zn) was found to be the predominant heavy metal in S7 (Figure 2). A study conducted by Michalec and Cupak [56] observed the sources of As, Pb, and Cd in sediments found in small reservoirs located in southern Poland. These sources were found to be derived from agricultural watersheds. Furthermore, it has been demonstrated that the utilization of fertilizers and pesticides in farming procedures is responsible for the high level of arsenic (As) in the surface sediments of the Jialu River in China [57]. The concentrations of As, Pb, Hg, Ni, Cd, and Zn in sediments indicated significant relationships with the amount of fertilizer used within agricultural areas [58,59]. The main contributors of zinc (Zn) were identified as motor vehicles and untreated residential wastewater originating from community areas [60]. Additionally, it should be noted that the primary origins of Ni, Co, and Cr can be attributed to natural sources [51,58].

Principal component analysis (PCA) has been implemented as a technique to assess statistical elements related to soil contamination, resource exploitation, protection, and degradation of the environment [61,62]. PCA is a multivariate statistical methodology performed to identify and classify comparable variables based on their relationships. Table 3 illustrates the results of the PCA classification according to the sources of heavy metals found in the surface sediment of the Mae Chaem River. Loadings with a value exceeding 0.50 were considered statistically significant and indicated in bold. The observation of elevated levels of heavy metals in the surface sediment of the Mae Chaem River can be attributed to two distinct factors. The cumulative variance of the first component at the receptor site was 48.7%, which demonstrates the high proportions of Cd, Cr, Ni, Cu, and Zn. The previous features indicate the presence of contamination within the community area. Balabanova et al. [62] suggest that urban areas represent the major source of As, Cd, Cu, and Pb. The second factor described a significant proportion of the general variance, approximately 77.8%. This factor displayed high loadings of As and Pb, which are commonly associated with agricultural areas [58,59]. According to Zhou et al. (2021), the presence of As and Cd in eastern China can be attributed to agricultural and industrial activities [63]. Therefore, a link can exist between the surface sediments of the Mae Chaem River and the surrounding communities and agricultural areas.

### 3.2. Assessment of Surface Sediment Pollution for Mae Chaem River

#### 3.2.1. Geo-Accumulation (I*_geo_*)

**Figure 3:**
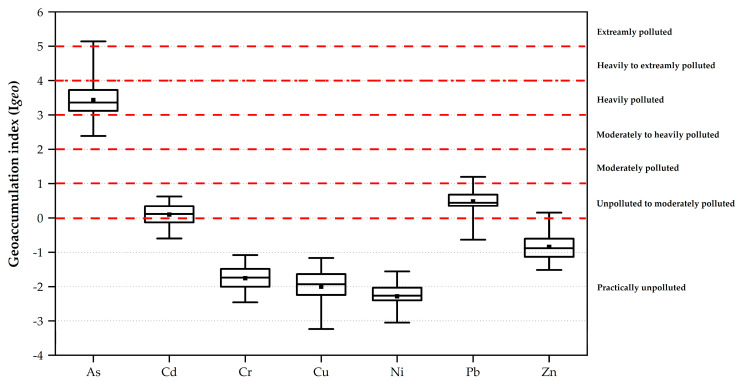
Box boxplot of I*_geo_* value of heavy metals in the sediment of Mae Chaem River.

Figure 3 shows a boxplot representing the mean I*_geo_* values for heavy metals found in the surface sediment of the Mae Chaem River, Chiang Mai, Thailand. The I*_geo_* values show a range of 2.39 to 5.14 for As, −0.60 to 0.62 for Cd, −2.46 to −1.08 for Cr, −3.05 to −1.56 for Ni, −0.64 to 1.20 for Pb, −3.24 to −1.17 for Cu, and −1.52 to 0.15 for Zn. The mean I*_geo_* values of As, Cd, Cr, Ni, Pb, Cu, and Zn were 3.25, 0.09, −2.27, 0.34, −2.00, and −0.84, respectively (Table S6). The pollution levels of Cr, Ni, Cu, and Zn are native values that indicate an unpolluted area, whereas Cd and Pb indicate an unpolluted to moderately polluted area. Arsenic was strongly observed in extremely polluted areas. Finally, the assessment of the I*_geo_* values of heavy metals in the surface sediment of the Mae Chaem River indicates that As may be accumulated in agricultural areas.

#### 3.2.2. Contamination Factor (CF) and Pollution Load Index (PLI)

Table 4 illustrates the values of the contamination factor (CF) and pollution load index (PLI). The heavy metal concentrations in surface sediments of the Mae Chaem River were evaluated using the CF method. The CF values, arranged in descending order, were as follows: As (2.17) > Cd (1.64) > Ni (0.92) > Pb (0.78) > Cu (0.62) Zn (0.61) > Cr (0.51). The results indicate that the CF of As and Cd are indicative of a moderate degree of pollution, falling within the range of 1 to 3. On the other hand, the CF values for Nickel (Ni), Lead (Pb), Copper (Cu), Zinc (Zn), and Chromium (Cr) show a lower degree of pollution, with CF values below 1.

Additionally, the investigation of surface sediments obtained from S4 reveals that the CF value for As was recorded as 6.34, exceeding the acceptable limit of 6. This observation suggests the presence of exceedingly elevated pollution levels (CF > 6). The present study addressed the occurrence of elevated levels of arsenic (As) in sediment on agricultural land, which was attributed to the use of pesticides that were frequently used in agricultural practices [36,51,64]. The monitoring report conducted on the Mae Chaem River over the period of July 2020 to August 2021 indicated that the concentration of As (0.02 mg/L) exceeded the established Thai surface water standard (0.01 mg/L) [65].

**Figure 4:**
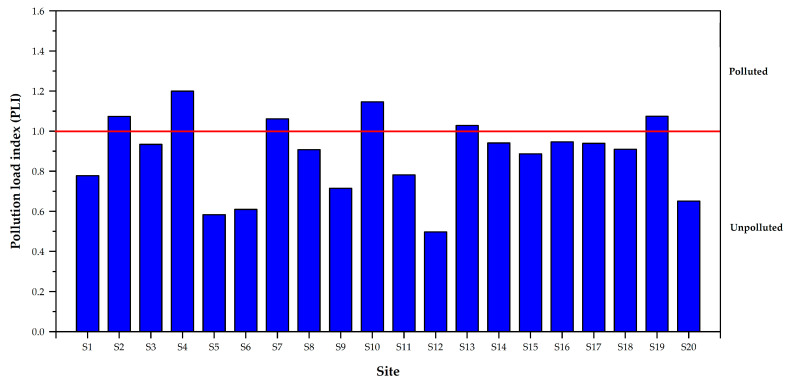
Pollution load index (PLI) classification in the sediment of Mae Chaem River.

The PLI values presented an assessment of the total toxicity level of the sample due to heavy metal contribution (Figure 4). The PLI values of heavy metals in surface sediments in the Mae Chaem River ranged from 0.50 to 1.20, with a mean PLI value of 0.88 ± 0.20, indicating no metal contamination in the sediment (PLI < 1.0). PLI values for S2, S4, S7, S10, S13, and S19 in this study were 1.07, 1.20, 1.06, 1.15, 1.03, and 1.07, indicating PLI > 1.0 and implying pollution. Agricultural areas dominate the majority of the Mae Chaem River area.

### 3.3. Health Risk Assessment for Metals in Surface Sediments of the Mae Chaem River

#### 3.3.1. Non-Carcinogenic Risk by Hazard Quotient (HQ)

The assessment of non-carcinogenic risk associated with exposure to heavy-metal-bound surface sediments in the Mae Chaem River in Chiang Mai Province was conducted by using HQ values. Non-carcinogenic risk refers to any unsuitable effects on health other than cancer in an organism, caused by factors in the environment. HQ can be determined as the calculated ratio of the average daily dose (ADI) and the reference values (RfD). Table 5 presents the results from the estimation of non-carcinogenic risks associated with ingesting heavy metals from surface sediment in the Mae Chaem River. HQ values for children affected by the intake of heavy metals in the surface sediment of the Mae Chaem River were observed to range from 6.02 × 10^−6^ to 4.05 × 10^−1^ for As, 2.53 × 10^−4^ to 5.90 × 10^−4^ for Cd, 1.16 × 10^−2^ to 3.02 × 10^−2^ for Cr, 1.58 × 10^−3^ to 4.43 × 10^−3^ for Ni, 4.41 × 10^−3^ to 1.57 × 10^−2^ for Pb, 2.80 × 10^−4^ to 1.18 × 10^−3^ for Cu, and 1.56 × 10^−4^ to 4.97 × 10^−4^ for Zn, while the HQ levels of As, Cd, Cr, Ni, Pb, Cu, and Zn for adults were 6.45 × 10^−3^ to 4.34 × 10^−2^, 2.71 × 10^−5^ to 6.33 × 10^−5^, 1.25 × 10^−3^ to 3.23 × 10^−3^, 1.69 × 10^−4^ to 4.75 × 10^−4^, 4.72 × 10^−4^ to 1.68 × 10^−3^, 3.00 × 10^−5^ to 1.26 × 10^−5^, and 1.67 × 10^−5^ to 5.33 × 10^−5^, respectively. However, the hazard quotient (HQ) values corresponding to the exposure to heavy metals from the surface sediment of the Mae Chaem River were found to be below 1.0 (HQ < 1.0) for both children and adults. This observation suggests that the risk linked to exposure is low. The HQ values of exposure to ingestion of heavy metals by individuals from the surface sediment of the Mae Chaem River, as determined for both children and adults, were categorized in descending order as follows: As > Cr > Pb > Ni > Cu > Cd > Zn. The results were consistent with Li et al.’s (2022) study, indicating that individuals of all age groups showed HQ values below 1.0 when exposed to heavy metals from soil and ditch sediments in a long-term mining waste area. Particularly, children were shown to be more sensitive in comparison with adults.

In a study conducted by Gunes (2022), it was shown that the hazard quotient (HQ) values associated with the intake of heavy metals from sediment in Turkey’s Bartin River increased for children and adults during both the dry and wet seasons. Specifically, the HQ values ranged from 1.26 × 10^−1^ to 4.66 × 10^−1^ for children and adults in the rainy season, and from 4.27 × 10^−2^ to 1.60 × 10^−1^ in the dry season. However, the value was found to be below 1.0, suggesting an acceptable level of risk (HQ < 1.0) [44]. Moreover, the study conducted by Hasaballah et al. (2022) revealed the order of cumulative hazard quotient (HQ) values associated with heavy metal exposure in the Nile River, Damietta, Egypt, as follows: Cd > Pb > Co > Ni > Zn > Cu > Fe. The toxicity of heavy metals has been suggested to be relevant for the reported HQ values exceeding 1.0 [47].

#### 3.3.2. Carcinogenic Risk by the Lifetime Cancer Risk (LCR)

Table 6 shows the data on lifetime cancer risk (LCR) used to evaluate the long-term effects of heavy metals, assuming a lifetime carcinogenic risk to human inhalation for 70 years. Based on specific theoretical frameworks, it has been suggested that the presence of poisonous heavy metals including As, Cd, Cr, Ni, and Pb may be causally linked to the occurrence of cancer [49]. The mean LCR values in the surface sediment of the Mae Chaem River were determined for two age groups, children and adults, as the following: As, Cd, Cr, Ni, and Pb were 9.94 × 10^−7^ to 1.56 × 10^−5^, 5.85 × 10^−8^ to 3.19 × 10^−7^, 6.40 × 10^−7^ to 3.88 × 10^−6^, 5.74 × 10^−11^ to 3.76 × 10^−10^, and 4.82 × 10^−9^ to 4.01 × 10^−8^, respectively. However, the levels of LCR associated with the ingestion of carcinogenic metals found in the surface sediment of the Mae Chaem River indicate a situation of low risk, with a value less than 10^−6^. The societal lifetime cancer risk (cases/million population) was estimated by multiplying the LCR by one million citizens. The societal LCR values of long-term toxic metal exposure from surface sediment of the Mae Chaem River for children and adults were 5 and 2 cases/people for As, 0.2 and 0.1 cases/people for Cd, 2 and 1 cases/people for Cr, 0.0002 and 0.0001 cases/people for Ni, and 0.02 and 0.01 cases/people for Pb. The vulnerability of children’s anatomy and physiology to environmental contaminants, however, may potentially be different to that of adults due to toxicodynamic (e.g., when exposures take place during times of increased susceptibility) and/or toxicokinetic changes (i.e., differences in absorption, metabolism, and excretion). The behavior of children and their activity levels are different from those of adults, and they can be exposed to pollutants and other toxins while engaging in typical oral exploration of their surroundings (i.e., hand-to-mouth behavior) and by touching objects such as toys, floors, and surfaces (US-EPA, 2011). As a result, it is possible that children living in agricultural areas who participate in recreational activities in nearby fields located in the Mae Chaem District of Chiang Mai Province may be exposed to As and Cr at higher levels than other children.

## 4. Conclusions

We reported that the concentrations of heavy metals in the sediment samples from the Mae Chaem River in Chiang Mai, Thailand, were investigated during the dry season in 2021. The mean concentrations of heavy metals in the sediments were Zn > Cr > As > Pb > Ni > Cu > Cd. The levels of As, Cd, Cu, and Cr were higher than the national standard in Thailand. However, they were lower than the limits set by the WHO. The hazard quotient (HQ) values for heavy metal exposure from the surface sediment of the Mae Chaem River were less than 1.0, indicating a low level of concern in terms of potential hazards. The lifetime cancer risk (LCR) associated with this environment was classified as low, with a value below 10^−6^. However, it is possible that children living in agricultural areas may be exposed to higher levels of As and Cr. To our knowledge, the present study is the first project investigating this area, which is one of the intensive areas of agricultures in northern Thailand. Therefore, effective mitigation strategies in response to metal contamination relevant to the area are required to protect the health of local communities.

## Figures and Tables

**Table 1:** The concentration of heavy metals (mg Kg^−1^) in the surface sediments of the Mae Chaem River.

Site	As	Cd	Cr	Ni	Pb	Cu	Zn
S1	30.0	0.27	44.4	21.7	23.8	18.5	43.7
S2	27.2	0.42	58.3	36.1	24.0	36.8 *	67.2
S3	40.9	0.40	37.3	23.5	28.6	26.0	50.6
S4	95.1 *	0.35	38.0	29.8	39.5	35.8	58.1
S5	14.1	0.28	27.3	13.6	24.9	11.2	48.0
S6	15.9	0.27	28.6	13.8	25.9	12.5	50.1
S7	46.9	0.32	45.4	21.5	43.0	17.9	117 *
S8	58.9	0.28	30.2	21.8	33.3	21.4	55.9
S9	26.6	0.27	43.6	21.2	21.2	13.9	42.3
S10	30.3	0.43	64.7	38.1 *	31.5	28.0	78.8
S11	28.2	0.34	43.8	21.4	20.1	17.5	47.8
S12	16.4	0.20	34.6	14.9	12.1	8.75	36.7
S13	38.9	0.40	53.2	27.8	28.6	24.9	64.5
S14	18.3	0.35	70.8 *	25.8	25.7	21.7	86.5
S15	31.6	0.31	49.4	25.1	24.0	22.1	56.6
S16	25.5	0.32	53.7	27.1	27.1	26.0	70.7
S17	32.9	0.33	59.7	27.4	25.0	21.6	57.3
S18	25.1	0.37	45.6	23.2	25.2	27.0	67.0
S19	22.9	0.46 *	50.6	28.5	40.1 *	28.3	81.9
S20	23.9	0.22	37.5	16.9	19.7	17.3	38.4
**Mean**	32.5	0.33	45.8	24.0	27.2	21.9	60.9
**S.D.**	18.3	0.07	11.9	7.50	6.56	7.42	19.3
**Range**	14.1–95.5	0.20–0.46	27.3–70.8	13.6–38.1	12.1–43.0	8.75–36.8	36.7–116.7
**PCD**	10.0	0.16	45.5	27.5	36.0	21.5	80.0
**WHO**	N.D.	0.99	43.4	22.7	35.8	31.6	121.0

* The highest concentrations of any individual heavy metal. PCD = Pollution Control Department, Thailand. WHO = World Health Organization.

**Table 2:** Comparison of heavy metal concentrations found in other studies and this study.

Study Area	Mean Concentrations of Heavy Metal in the Sediment (mg Kg^−1^)							References
	As	Cd	Cr	Ni	Pb	Cu	Zn	
Mae Chaem River, Chiang Mai, Thailand	32.5	0.33	45.8	24.0	27.2	21.9	60.9	This study
Batin River, Batin Province, Turkey	4.0	NA	19.0	24.7	14.0	27.0	57.5	[44]
Xiangjiang River, China	98.4	23.3	59.7	36.3	102.5	71.3	258	[51]
Shitalakhya River, Bangladesh	NA	0.43	10.2	29.1	24.7	54.4	51.0	[52]
Majiagou River, Habin, China	NA	0.76	107	17.8	26.9	28.1	358	[50]
Yunliang River, Habin, China	NA	1.83	68.2	8.16	32.8	19.5	861	[50]
Lam Plai Mat River, Buriram Province, Thailand	NA	0.17	NA	NA	12.4	4.4	19.2	[54]
Chao Phraya River, Bangkok, Thailand	NA	0.13–0.22	NA	NA	15.1–28.8	NA	21.9–103	[55]
Mae Klong River, Samut Songkhram Province, Thailand	NA	ND	NA	NA	1.32	0.61	1.99	[53]

**Table 3:** Factor loading of the seven heavy metals analyzed in sediment samples (n = 20).

Heavy Metal	Component	
	1	2
As	0.002	**0.903**
Cd	**0851**	0.263
Cr	**0.898**	−0.260
Ni	**0.902**	0.228
Pb	0.347	**0.830**
Cu	**0.737**	0.480
Zn	**0.639**	0.325
Eigenvalues	3.97	1.46
% Of variance	48.7	28.9
Cumulative %	48.7	77.8
Estimate sources	Community area	Agriculture area

Factor loading values of >0.50 are shown in bold.

**Table 4:** Contamination factor (CF) and pollution load index (PLI).

Sites	CF Value							PLI Value
	As	Cd	Cr	Ni	Pb	Cu	Zn	
S1	2.00	1.33	0.49	0.84	0.68	0.53	0.44	0.78
S2	1.81	2.08	0.65	1.39	0.69	1.05	0.67	1.07
S3	2.73	1.99	0.41	0.90	0.82	0.74	0.51	0.93
S4	6.34	1.74	0.42	1.15	1.13	1.02	0.58	1.20
S5	0.94	1.41	0.30	0.52	0.71	0.32	0.48	0.58
S6	1.06	1.33	0.32	0.53	0.74	0.36	0.50	0.61
S7	3.13	1.58	0.50	0.83	1.23	0.51	1.17	1.06
S8	3.92	1.41	0.34	0.84	0.95	0.61	0.56	0.91
S9	1.77	1.33	0.48	0.82	0.61	0.40	0.42	0.71
S10	2.02	2.15	0.72	1.47	0.90	0.80	0.79	1.15
S11	1.88	1.72	0.49	0.82	0.57	0.50	0.48	0.78
S12	1.09	0.99	0.38	0.57	0.34	0.25	0.37	0.50
S13	2.59	1.98	0.59	1.07	0.82	0.71	0.65	1.03
S14	1.22	1.74	0.79	0.99	0.73	0.62	0.87	0.94
S15	2.10	1.57	0.55	0.97	0.69	0.63	0.57	0.89
S16	1.70	1.58	0.60	1.04	0.78	0.74	0.71	0.95
S17	2.19	1.66	0.66	1.05	0.71	0.62	0.57	0.94
S18	1.67	1.83	0.51	0.89	0.72	0.77	0.67	0.91
S19	1.53	2.31	0.56	1.10	1.14	0.81	0.82	1.07
S20	1.59	1.08	0.42	0.65	0.56	0.49	0.38	0.65
**Mean**	**2.17**	**1.64**	**0.51**	**0.92**	**0.78**	**0.62**	**0.61**	**0.88**
**S.D.**	**1.22**	**0.35**	**0.13**	**0.25**	**0.21**	**0.21**	**0.19**	**0.20**

**Table 5:** Non-carcinogenic risk of heavy metals in sediments in Mae Chaem River calculated by the hazard quotient (HQ).

Site	HQ for Children							HQ for Adults						
	As	Cd	Cr	Ni	Pb	Cu	Zn	As	Cd	Cr	Ni	Pb	Cu	Zn
S1	1.3 × 10^−1^	3.4 × 10^−4^	1.9 × 10^−2^	2.5 × 10^−3^	8.7 × 10^−3^	5.9 × 10^−4^	1.9 × 10^−4^	1.4 × 10^−2^	3.6 × 10^−5^	2.0 × 10^−3^	2.7 × 10^−4^	9.3 × 10^−4^	6.3 × 10^−5^	2.0 × 10^−5^
S2	1.2 × 10^−1^	5.3 × 10^−4^	2.5 × 10^−2^	4.2 × 10^−3^	8.8 × 10^−3^	1.2 × 10^−3^	2.9 × 10^−4^	1.2 × 10^−2^	5.7 × 10^−5^	2.7 × 10^−3^	4.5 × 10^−4^	9.4 × 10^−4^	1.3 × 10^−4^	3.1 × 10^−5^
S3	1.7 × 10^−1^	5.1 × 10^−4^	1.6 × 10^−2^	2.7 × 10^−3^	1.0 × 10^−2^	8.3 × 10^−4^	2.2 × 10^−4^	1.9 × 10^−2^	5.4 × 10^−5^	1.7 × 10^−3^	2.9 × 10^−4^	1.1 × 10^−3^	8.9 × 10^−5^	2.3 × 10^−5^
S4	4.1 × 10^−1^	4.5 × 10^−4^	1.6 × 10^−2^	3.5 × 10^−3^	1.4 × 10^−2^	1.1 × 10^−3^	2.5 × 10^−4^	4.3 × 10^−2^	4.8 × 10^−5^	1.7 × 10^−3^	3.7 × 10^−4^	1.5 × 10^−3^	1.2 × 10^−4^	2.7 × 10^−5^
S5	6.0 × 10^−2^	3.6 × 10^−4^	1.2 × 10^−2^	1.6 × 10^−3^	9.1 × 10^−3^	3.6 × 10^−4^	2.0 × 10^−4^	6.4 × 10^−3^	3.9 × 10^−5^	1.2 × 10^−3^	1.7 × 10^−4^	9.8 × 10^−4^	3.8 × 10^−5^	2.2 × 10^−5^
S6	6.8 × 10^−2^	3.4 × 10^−4^	1.2 × 10^−2^	1.6 × 10^−3^	9.5 × 10^−3^	4.0 × 10^−4^	2.1 × 10^−4^	7.3 × 10^−3^	3.6 × 10^−5^	1.3 × 10^−3^	1.7 × 10^−4^	1.0 × 10^−3^	4.3 × 10^−5^	2.3 × 10^−5^
S7	2.0 × 10^−1^	4.0 × 10^−4^	1.9 × 10^−2^	2.5 × 10^−3^	1.6 × 10^−2^	5.7 × 10^−4^	5.0 × 10^−4^	2.1 × 10^−2^	4.3 × 10^−5^	2.1 × 10^−3^	2.7 × 10^−4^	1.7 × 10^−3^	6.1 × 10^−5^	5.3 × 10^−5^
S8	2.5 × 10^−1^	3.6 × 10^−4^	1.3 × 10^−2^	2.5 × 10^−3^	1.2 × 10^−2^	6.9 × 10^−4^	2.4 × 10^−4^	2.7 × 10^−2^	3.9 × 10^−5^	1.4 × 10^−3^	2.7 × 10^−4^	1.3 × 10^−3^	7.3 × 10^−5^	2.6 × 10^−5^
S9	1.1 × 10^−1^	3.4 × 10^−4^	1.9 × 10^−2^	2.5 × 10^−3^	7.8 × 10^−3^	4.4 × 10^−4^	1.4 × 10^−4^	1.2 × 10^−2^	3.7 × 10^−5^	2.0 × 10^−3^	2.6 × 10^−4^	8.4 × 10^−4^	4.8 × 10^−5^	1.9 × 10^−5^
S10	1.3 × 10^−1^	5.5 × 10^−4^	2.8 × 10^−2^	4.4 × 10^−3^	1.2 × 10^−2^	8.9 × 10^−4^	3.4 × 10^−4^	1.4 × 10^−2^	5.9 × 10^−5^	3.0 × 10^−3^	4.8 × 10^−4^	1.2 × 10^−3^	9.6 × 10^−5^	3.6 × 10^−5^
S11	1.2 × 10^−1^	4.4 × 10^−4^	1.9 × 10^−2^	2.5 × 10^−3^	7.4 × 10^−3^	5.6 × 10^−4^	2.0 × 10^−4^	1.3 × 10^−2^	4.7 × 10^−5^	2.0 × 10^−3^	2.7 × 10^−4^	7.9 × 10^−4^	6.0 × 10^−5^	2.2 × 10^−5^
S12	7.0 × 10^−2^	2.5 × 10^−4^	1.5 × 10^−2^	1.7 × 10^−3^	4.4 × 10^−3^	2.8 × 10^−4^	1.6 × 10^−4^	7.5 × 10^−3^	2.7 × 10^−5^	1.6 × 10^−3^	1.9 × 10^−4^	4.7 × 10^−4^	3.0 × 10^−5^	1.7 × 10^−5^
S13	1.7 × 10^−1^	5.1 × 10^−4^	2.3 × 10^−2^	3.2 × 10^−3^	1.0 × 10^−2^	8.0 × 10^−4^	2.8 × 10^−4^	1.8 × 10^−2^	5.4 × 10^−5^	2.4 × 10^−3^	3.5 × 10^−4^	1.1 × 10^−3^	8.5 × 10^−5^	2.9 × 10^−5^
S14	7.8 × 10^−2^	4.4 × 10^−4^	3.0 × 10^−2^	3.0 × 10^−3^	9.4 × 10^−3^	6.9 × 10^−4^	3.7 × 10^−4^	8.3 × 10^−3^	4.8 × 10^−5^	3.2 × 10^−3^	3.2 × 10^−4^	1.0 × 10^−3^	7.4 × 10^−5^	4.0 × 10^−5^
S15	1.3 × 10^−1^	4.0 × 10^−4^	2.1 × 10^−2^	2.9 × 10^−3^	8.8 × 10^−3^	7.1 × 10^−4^	2.4 × 10^−4^	1.4 × 10^−2^	4.3 × 10^−5^	2.3 × 10^−3^	3.1 × 10^−4^	9.4 × 10^−4^	7.6 × 10^−5^	2.6 × 10^−5^
S16	1.1 × 10^−1^	4.0 × 10^−4^	2.3 × 10^−2^	3.2 × 10^−3^	9.9 × 10^−3^	8.3 × 10^−4^	3.0 × 10^−4^	1.2 × 10^−2^	4.3 × 10^−5^	2.5 × 10^−3^	3.4 × 10^−4^	1.1 × 10^−3^	8.9 × 10^−5^	3.2 × 10^−5^
S17	1.4 × 10^−1^	4.2 × 10^−4^	2.5 × 10^−2^	3.2 × 10^−3^	9.1 × 10^−3^	6.9 × 10^−4^	2.4 × 10^−4^	1.5 × 10^−2^	4.6 × 10^−5^	2.7 × 10^−3^	3.4 × 10^−4^	9.8 × 10^−4^	7.4 × 10^−5^	2.6 × 10^−5^
S18	1.1 × 10^−1^	4.7 × 10^−4^	1.9 × 10^−2^	2.7 × 10^−3^	9.2 × 10^−3^	8.6 × 10^−4^	2.9 × 10^−4^	1.1 × 10^−2^	5.0 × 10^−5^	2.1 × 10^−3^	2.9 × 10^−4^	9.9 × 10^−4^	9.3 × 10^−5^	3.1 × 10^−5^
S19	9.8 × 10^−2^	5.9 × 10^−4^	2.2 × 10^−2^	3.3 × 10^−3^	1.5 × 10^−2^	9.1 × 10^−4^	3.5 × 10^−4^	1.0 × 10^−2^	6.3 × 10^−5^	2.3 × 10^−3^	3.5 × 10^−4^	1.6 × 10^−3^	9.7 × 10^−5^	3.7 × 10^−5^
S20	1.0 × 10^−1^	2.8 × 10^−4^	1.6 × 10^−2^	2.0 × 10^−3^	7.2 × 10^−3^	5.5 × 10^−4^	1.6 × 10^−4^	1.1 × 10^−2^	2.9 × 10^−5^	1.7 × 10^−3^	2.1 × 10^−4^	7.7 × 10^−4^	5.9 × 10^−5^	1.8 × 10^−5^
**Mean**	1.4 × 10^−1^	4.2 × 10^−4^	2.0 × 10^−2^	2.8 × 10^−3^	9.9 × 10^−3^	7.0 × 10^−4^	2.6 × 10^−4^	1.5 × 10^−2^	4.5 × 10^−5^	2.1 × 10^−3^	3.0 × 10^−4^	1.1 × 10^−3^	7.5 × 10^−5^	2.8 × 10^−5^
**Min**	6.0 × 10^−2^	2.5 × 10^−4^	1.2 × 10^−2^	1.6 × 10^−3^	4.4 × 10^−3^	2.8 × 10^−4^	1.6 × 10^−4^	6.4 × 10^−3^	2.7 × 10^−5^	1.2 × 10^−3^	1.7 × 10^−4^	4.7 × 10^−4^	3.0 × 10^−5^	1.7 × 10^−5^
**Max**	4.1 × 10^−1^	5.9 × 10^−4^	3.0 × 10^−2^	4.4 × 10^−3^	1.6 × 10^−2^	1.2 × 10^−3^	5.0 × 10^−3^	4.3 × 10^−2^	6.3 × 10^−5^	3.2 × 10^−3^	4.8 × 10^−4^	1.7 × 10^−3^	1.3 × 10^−4^	5.3 × 10^−5^

**Table 6:** Carcinogenic risk of toxic metals in sediments in Mae Chaem River calculated by the lifetime cancer risk (LCR).

Site	LCR for Children					LCR for Adults				
	As	Cd	Cr	Ni	Pb	As	Cd	Cr	Ni	Pb
S1	4.9 × 10^−6^	1.8 × 10^−7^	2.4 × 10^−6^	2.1 × 10^−10^	2.2 × 10^−8^	2.1 × 10^−6^	7.9 × 10^−8^	1.0 × 10^−6^	9.2 × 10^−11^	9.5 × 10^−9^
S2	4.5 × 10^−6^	2.9 × 10^−7^	3.2 × 10^−6^	3.6 × 10^−10^	2.2 × 10^−8^	1.9 × 10^−6^	1.2 × 10^−7^	1.4 × 10^−6^	1.5 × 10^−10^	9.6 × 10^−9^
S3	6.7 × 10^−6^	2.7 × 10^−7^	2.0 × 10^−6^	2.3 × 10^−10^	2.7 × 10^−8^	2.9 × 10^−6^	1.2 × 10^−7^	8.7 × 10^−7^	9.9 × 10^−11^	1.1 × 10^−8^
S4	1.6 × 10^−5^	2.4 × 10^−7^	2.1 × 10^−6^	2.9 × 10^−10^	3.7 × 10^−8^	6.7 × 10^−6^	1.0 × 10^−7^	8.9 × 10^−7^	1.3 × 10^−10^	1.6 × 10^−8^
S5	2.3 × 10^−6^	1.9 × 10^−7^	1.5 × 10^−6^	1.3 × 10^−10^	2.3 × 10^−8^	9.9 × 10^−7^	8.3 × 10^−8^	6.4 × 10^−7^	5.7 × 10^−11^	1.0 × 10^−8^
S6	2.6 × 10^−6^	1.8 × 10^−7^	1.6 × 10^−6^	1.4 × 10^−10^	2.4 × 10^−8^	1.1 × 10^−6^	7.9 × 10^−8^	6.7 × 10^−7^	5.8 × 10^−11^	1.0 × 10^−8^
S7	7.7 × 10^−6^	2.2 × 10^−7^	2.5 × 10^−6^	2.1 × 10^−10^	4.0 × 10^−8^	3.3 × 10^−6^	9.4 × 10^−8^	1.1 × 10^−6^	9.1 × 10^−11^	1.7 × 10^−8^
S8	9.7 × 10^−6^	1.9 × 10^−7^	1.7 × 10^−6^	2.1 × 10^−10^	3.1 × 10^−8^	4.1 × 10^−6^	8.4 × 10^−8^	7.1 × 10^−7^	9.2 × 10^−11^	1.3 × 10^−8^
S9	4.4 × 10^−6^	1.8 × 10^−7^	2.4 × 10^−6^	2.1 × 10^−10^	2.0 × 10^−8^	1.9 × 10^−6^	7.9 × 10^−8^	1.0 × 10^−6^	9.0 × 10^−11^	8.5 × 10^−9^
S10	5.0 × 10^−6^	3.0 × 10^−7^	3.5 × 10^−6^	3.8 × 10^−10^	2.9 × 10^−8^	2.1 × 10^−6^	1.3 × 10^−7^	1.5 × 10^−6^	1.6 × 10^−10^	1.3 × 10^−8^
S11	4.6 × 10^−6^	2.4 × 10^−7^	2.4 × 10^−6^	2.1 × 10^−10^	1.9 × 10^−8^	2.0 × 10^−6^	1.0 × 10^−7^	1.0 × 10^−6^	9.0 × 10^−11^	8.0 × 10^−9^
S12	2.7 × 10^−6^	1.4 × 10^−7^	1.9 × 10^−6^	1.5 × 10^−10^	1.1 × 10^−8^	1.2 × 10^−6^	5.9 × 10^−8^	8.1 × 10^−7^	6.3 × 10^−11^	4.8 × 10^−9^
S13	6.4 × 10^−6^	2.7 × 10^−7^	2.9 × 10^−6^	2.7 × 10^−10^	2.7 × 10^−8^	2.7 × 10^−6^	1.2 × 10^−7^	1.2 × 10^−6^	1.2 × 10^−10^	1.1 × 10^−8^
S14	3.0 × 10^−6^	2.4 × 10^−7^	3.9 × 10^−6^	2.5 × 10^−10^	2.4 × 10^−8^	1.3 × 10^−6^	1.0 × 10^−7^	1.7 × 10^−6^	1.1 × 10^−10^	1.0 × 10^−8^
S15	5.2 × 10^−6^	2.2 × 10^−7^	2.7 × 10^−6^	2.5 × 10^−10^	2.2 × 10^−8^	2.2 × 10^−6^	9.3 × 10^−8^	1.2 × 10^−6^	1.1 × 10^−10^	9.6 × 10^−9^
S16	4.2 × 10^−6^	2.2 × 10^−7^	2.9 × 10^−6^	2.7 × 10^−10^	2.5 × 10^−8^	1.8 × 10^−6^	9.4 × 10^−8^	1.3 × 10^−6^	1.1 × 10^−10^	1.1 × 10^−8^
S17	5.4 × 10^−6^	2.3 × 10^−7^	3.3 × 10^−6^	2.7 × 10^−10^	2.3 × 10^−8^	2.3 × 10^−6^	9.8 × 10^−8^	3.3 × 10^−6^	1.2 × 10^−10^	1.0 × 10^−8^
S18	4.1 × 10^−6^	2.5 × 10^−7^	2.5 × 10^−6^	2.3 × 10^−10^	2.4 × 10^−8^	1.8 × 10^−6^	1.1 × 10^−7^	1.4 × 10^−6^	9.8 × 10^−11^	1.0 × 10^−8^
S19	3.8 × 10^−6^	3.2 × 10^−7^	2.8 × 10^−6^	2.8 × 10^−10^	3.7 × 10^−8^	1.6 × 10^−6^	1.4 × 10^−7^	1.2 × 10^−6^	1.2 × 10^−10^	1.6 × 10^−8^
S20	3.9 × 10^−6^	1.5 × 10^−7^	2.1 × 10^−6^	1.7 × 10^−10^	1.8 × 10^−8^	1.7 × 10^−6^	6.4 × 10^−8^	8.8 × 10^−7^	7.1 × 10^−11^	7.9 × 10^−9^
**Mean**	5.3 × 10^−6^	2.3 × 10^−7^	2.5 × 10^−6^	2.4 × 10^−10^	2.5 × 10^−8^	2.3 × 10^−6^	9.7 × 10^−8^	1.1 × 10^−6^	1.0 × 10^−10^	1.1 × 10^−8^
**S.D.**	3.0 × 10^−6^	4.9 × 10^−8^	6.5 × 10^−7^	6.5 × 10^−11^	6.9 × 10^−9^	1.3 × 10^−6^	2.1 × 10^−8^	2.8 × 10^−7^	2.8 × 10^−11^	3.0 × 10^−9^
**Min**	2.3 × 10^−6^	1.4 × 10^−7^	1.5 × 10^−6^	1.3 × 10^−10^	1.1 × 10^−8^	9.9 × 10^−7^	5.9 × 10^−8^	6.4 × 10^−7^	5.7 × 10^−11^	4.8 × 10^−9^
**Max**	1.5 × 10^−5^	3.2 × 10^−7^	3.9 × 10^−6^	3.8 × 10^−10^	4.0 × 10^−8^	6.7 × 10^−5^	1.4 × 10^−7^	1.7 × 10^−6^	1.6 × 10^−10^	1.7 × 10^−8^

## Data Availability

The data that support the findings of this study are available from the corresponding author on reasonable request.

## Supplementary Materials

The following supporting information can be downloaded at: https://www.mdpi.com/article/10.3390/toxics11090780/s1, Table S1: Basic information about each sampling site of the Mae Chaem River. Table S2: Precision and accuracy data based on the extraction of the urban dust SRM1648a. Table S3: Concentration categories are based on a geo-accumulation index (I*_geo_*) and contamination factor (CF). Table S4: Parameters of the average daily intake (ADD) for metals. Table S5: Reference dose (RfD) and the cancer slope factor (CSF_oral_) for elements. Table S6: The index of geo-accumulation represents the heavy metal concentration in surface sediments.
