# Supplementary material for: Risk Assessment of Heavy Metals in Sediment Samples from the Mae Chaem River, Chiang Mai, Thailand
Source: Toxics. 2023 Sep 14;11(9):780. doi: 10.3390/toxics11090780 (PMC10534551; doi:10.3390/toxics11090780)
Supplement: Supplementary file 1 [file toxics-11-00780-s001.zip › toxics-2599154-supplementary.pdf]

SUPPORTING INFORMATION TO :

Risk Assessment of heavy metal in surface sediment samples from the Mae Chaem River, Chiang Mai, Thailand

Sawaeng Kawichai [1], Tippawan Prapamontol [1,*], Teetawat Santijitpakdee [1] and Susira Bootdee [2]

[1] Research Institute for Health Sciences (RIHES), Chiang Mai University, Chiang Mai 50200, Thailand; sawaeng.k@gmail.com (S.K.); teetawat_san@cmu.ac.th (T.S.)
[2] Faculty of Science, Energy, and Environment, King Mongkut's University of Technology North Bangkok (Rayong Campus), Rayong, 21120, Thailand; susira.b@sciee.kmutnb.ac.th
* Correspondence: tippawan.prapamontol@cmu.ac.th

Table S1. Basic information about each sampling site of the Mae Chaem River.

Site Number	Type	Latitude	Longitude	Attitude (m)	Major anthropogenic activity of the site
S1	Agricultural area	18.6789	98.3425	513	Agricultural activities
S2	Community area	18.6813	98.3389	516	Village area
S3	Community area	18.6839	98.3760	520	Village area
S4	Agricultural area	18.6656	98.3825	448	Agricultural activities
S5	Agricultural area	18.6445	98.3767	472	Agricultural runoff and Village area
S6	Community area	18.6416	98.3767	517	Village area
S7	Community area	18.5523	98.3944	556	Village area
S8	Agricultural area	18.5466	98.3829	512	Agricultural activities
S9	Agricultural area	18.5408	98.3524	454	Agricultural activities
S10	Community area	18.5411	98.3512	456	Village area
S11	Agricultural area	18.5123	98.3561	412	Agricultural runoff and Village area
S12	Community area	18.5111	98.3539	441	Village area
S13	Agricultural area	18.4985	98.3615	436	Agricultural runoff and residential area (sub-urban)
S14	Community area	18.4943	98.3596	438	Commercial and residential area, downtown of Mae Chaem district (Urban)
S15	Agricultural area	18.4927	98.3630	432	Agricultural runoff and Village area
S16	Community area	18.4883	98.3666	441	Village area
S17	Community area	18.4817	98.3703	426	Village area
S18	Agricultural area	18.4802	98.3700	410	Agricultural runoff and Village area
S19	Agricultural area	18.4302	98.3837	514	Agricultural activities
S20	Community area	18.2765	98.3854	324	Village area

Table S2. Precision and accuracy data based on the extraction of the urban dust SRM1648a.

Metals	R^2	LOD (µg/L)	SRM1648a (n = 3)		
			*ref. std conc. mg Kg^{-1}	% Rec.	% R.S.D.
**As**	0.99996	1.59	115.5 ± 3.9	117.4 ± 1.64	1.39
**Cd**	0.99985	0.74	73.7 ± 2.3	110.0 ± 2.59	2.35
**Cr**	0.99990	0.87	402 ± 13	48.7 ± 0.01	0.02
**Ni**	0.99975	1.13	81.1 ± 6.8	101.4 ± 1.21	1.19
**Pb**	0.99990	1.22	0.655 ± 0.033	105.7 ± 2.01	1.91
**Cu**	0.99994	1.19	610 ± 70	94.6 ± 2.32	2.45
**Zn**	0.99990	2.61	4800 ± 270	91.1 ± 1.01	1.11

Furthermore, the authors used Urban Particulate Matter as a reference material, it would have been more appropriate to use a standard sediment reference material.

Table S3. Concentration categories are based on a geo-accumulation index (I_{geo}) and contamination factor (CF).

I_{geo}			CF		
**Class**	**Values**	**Classification**	**Level**	**Value**	**Classification**
0	<0	Unpolluted	1	CF < 1	Low pollution
1	0-1	Unpolluted to moderately polluted	2	1 ≤ CF ≤ 3	Moderate pollution
2	1-2	Moderately polluted	3	3 ≤ CF ≤ 6	Considerable pollution
3	2-3	Moderately to strongly polluted	4	CF > 6	Very high pollution
4	3-4	Strongly to extremely polluted			
5	>4	Extremely polluted			

Table S4 Parameters of the average daily intake (ADD) for metals.

Parameters	Exposed group	
	**Children**	**Adult**
Intake rate (IR)	200 mg/day	100 mg/day
Exposure frequency (EF)	350 days/year	350 days/year
Exposure duration (ED)	6 years	24 years
Averaging time (AT)<br>AT = ED ×365 days	70 ×365= 25,550 days<br>(Carcinogenic risk)<br>6 ×365= 2,190 days<br>(non-carcinogenic risk)	70 ×365= 25,550 days<br>(Carcinogenic risk)<br>24 ×365= 8,760 days<br>(Non-carcinogenic risk)
Bodyweight (BW)	15 kg	70 kg

Table S5 Reference dose (RfD) and the cancer slope factor (CSF_{oral}) for elements.

Elements	RfD (mg/Kg^{-1}day^{-1})	CSF_{oral} (mg/ Kg^{-1}day^{-1})$^{-1}$
As	0.0003	1.5
Cd	0.001	6.3
Cr[1]	0.003	0.5
Ni	0.011	9.0×10^{-5}
Pb	0.0035	0.0085
Cu	0.04	-
Zn	0.3	-

[1] CSF of Cr is represented by Hexavalent chromium (Cr (VI)), which has been classified to relate human cancer.

Table S6 The index of geo-accumulation represents the heavy metal concentration in surface sediments.

Site	I*geo* Value						
	**As**	**Cd**	**Cr**	**Cu**	**Ni**	**Pb**	**Zn**
**S1**	3.47	-0.18	-1.75	-2.15	-2.37	0.34	-1.27
**S2**	3.33	0.47	-1.36	-1.17	-1.64	0.36	-0.64
**S3**	3.92	0.40	-2.01	-1.67	-2.26	0.61	-1.05
**S4**	5.14	0.21	-1.98	-1.20	-1.92	1.07	-0.85
**S5**	2.39	-0.09	-2.46	-2.88	-3.05	0.41	-1.13
**S6**	2.56	-0.17	-2.39	-2.72	-3.03	0.47	-1.07
**S7**	4.12	0.08	-1.72	-2.20	-2.38	1.20	0.15
**S8**	4.45	-0.09	-2.31	-1.94	-2.37	0.83	-0.91
**S9**	3.30	-0.17	-1.78	-2.57	-2.41	0.18	-1.31
**S10**	3.49	0.52	-1.21	-1.56	-1.56	0.75	-0.41
**S11**	3.38	0.20	-1.78	-2.24	-2.40	0.10	-1.13
**S12**	2.60	-0.60	-2.11	-3.24	-2.92	-0.64	-1.52
**S13**	3.85	0.40	-1.49	-1.73	-2.02	0.61	-0.70
**S14**	2.76	0.21	-1.08	-1.93	-2.12	0.45	-0.28
**S15**	3.55	0.07	-1.60	-1.90	-2.16	0.36	-0.89
**S16**	3.24	0.08	-1.48	-1.67	-2.05	0.53	-0.57
**S17**	3.61	0.15	-1.33	-1.93	-2.04	0.41	-0.87
**S18**	3.22	0.28	-1.72	-1.61	-2.28	0.43	-0.65
**S19**	3.09	0.62	-1.57	-1.54	-1.98	1.10	-0.36
**S20**	3.15	-0.48	-2.00	-2.25	-2.73	0.07	-1.45
